# Complete Genome Sequence of *Treponema paraluiscuniculi*, Strain Cuniculi A: The Loss of Infectivity to Humans Is Associated with Genome Decay

**DOI:** 10.1371/journal.pone.0020415

**Published:** 2011-05-31

**Authors:** David Šmajs, Marie Zobaníková, Michal Strouhal, Darina Čejková, Shannon Dugan-Rocha, Petra Pospíšilová, Steven J. Norris, Tom Albert, Xiang Qin, Kym Hallsworth-Pepin, Christian Buhay, Donna M. Muzny, Lei Chen, Richard A. Gibbs, George M. Weinstock

**Affiliations:** 1 Department of Biology, Faculty of Medicine, Masaryk University, Brno, Czech Republic; 2 Human Genome Sequencing Center, Baylor College of Medicine, Houston, Texas, United States of America; 3 Department of Pathology and Laboratory Medicine, University of Texas Medical School at Houston, Houston, Texas, United States of America; 4 NimbleGen Systems, Inc., Madison, Wisconsin, United States of America; 5 The Genome Sequencing Center, Department of Genetics, Washington University School of Medicine, St. Louis, Missouri, United States of America; University of North Carolina at Charlotte, United States of America

## Abstract

*Treponema paraluiscuniculi* is the causative agent of rabbit venereal spirochetosis. It is not infectious to humans, although its genome structure is very closely related to other pathogenic *Treponema* species including *Treponema pallidum* subspecies *pallidum*, the etiological agent of syphilis. In this study, the genome sequence of *Treponema paraluiscuniculi*, strain Cuniculi A, was determined by a combination of several high-throughput sequencing strategies. Whereas the overall size (1,133,390 bp), arrangement, and gene content of the Cuniculi A genome closely resembled those of the *T. pallidum* genome, the *T. paraluiscuniculi* genome contained a markedly higher number of pseudogenes and gene fragments (51). In addition to pseudogenes, 33 divergent genes were also found in the *T. paraluiscuniculi* genome. A set of 32 (out of 84) affected genes encoded proteins of known or predicted function in the Nichols genome. These proteins included virulence factors, gene regulators and components of DNA repair and recombination. The majority (52 or 61.9%) of the Cuniculi A pseudogenes and divergent genes were of unknown function. Our results indicate that *T*. *paraluiscuniculi* has evolved from a *T. pallidum*-like ancestor and adapted to a specialized host-associated niche (rabbits) during loss of infectivity to humans. The genes that are inactivated or altered in *T. paraluiscuniculi* are candidates for virulence factors important in the infectivity and pathogenesis of *T. pallidum* subspecies.

## Introduction

*Treponema paraluiscuniculi* is a noncultivable species of the genus *Treponema* that causes venereal spirochetosis in rabbits, but that is not infectious to humans [1]. The genome structure and chromosome sequence of *T*. *paraluiscuniculi* is closely related to other pathogenic species and subspecies of the *Treponema* genus, including syphilis causing spirochete *Treponema pallidum* ssp. *pallidum* [2] and *Treponema pallidum* ssp. *pertenue*, the causative agent of yaws.

The presence of spirochetes resembling *T. pallidum* in rabbit genital lesions was reported as early as 1912, and organism was described as *Spirochaeta paralues-cuniculi* (syphilis-like spirochetes in rabbits) by Jacobsthal [3]. The naturally occurring infection of rabbits with *T. paraluiscuniculi* is described in a detailed historical review by Smith and Pesetsky [4], as well as in more recent articles by DiGiacomo et al. [5], [6]. The disease is typically sexually transmitted, and results in erythema and edema of the prepuce, vagina, anus, or scrotum, often followed by ulceration and crusting (eschar formation) of the lesion. Infection of the nose, eyelids, lips, and paws can also occur. Intradermal inoculation of rabbits with either *T*. *paraluiscuniculi* or *T. pallidum* results in erythematous lesions that may undergo ulceration [4]–[8]. *T. paraluiscuniculi* lesions are noted to be less indurated (raised and hardened) than *T. pallidum* lesions. *T. pallidum* subspecies and *T. paraluiscuniculi* are nearly indistinguishable in terms of morphology, antigen content, and physiology [8]–[10], consistent with the close genetic relationship among these organisms [2]. However, *T. paraluiscuniculi* and *T. pallidum* cause different diseases with different host specificity. Rabbit venereal spirochetosis is characterized by localized genital lesions, whereas human syphilis is a multistage, sexually transmitted disease with varied clinical manifestations. Syphilis treponemes can infect virtually any human tissue, causing gummatous, neurologic, and cardiovascular manifestations [11], [12]. While there is little evidence for systemic manifestations following *T. paraluiscuniculi* infection in rabbits, the organism can disseminate and be recovered from lymph nodes months after infection [4], [13].

Evidence to date indicates that *T. paraluiscuniculi* is not pathogenic to humans, indicating a basic difference between this organism and the *T. pallidum* subspecies. Two studies involving a total of three volunteers described experimental inoculation of humans [1], [14] with rabbit virulent strains of *T. paraluiscuniculi* with negative results. When injected intradermally into human volunteers, rabbit virulent strains of *T. paraluiscuniculi* caused only mild, local erythema and edema (without systemic effects) that disappeared after three weeks [1], [14]. In the Graves and Downes study [1], only a limited serological response in the human volunteer was observed. In contrast, intradermally inoculated rabbits exhibited prominent, long-lasting lesions and a strong serological response to *T. paraluiscuniculi*.

Partial immunological cross-protection between *T. paraluiscuniculi* and *T. pallidum* has been observed, as demonstrated by infecting rabbits with one species, inoculating intradermally with the other species 3–6 months later, and observing lesion development for signs of decreased lesion frequency, severity, or duration [8]. Both serologic and T-cell reactivity indicate antigenic relatedness between these species [8], [9], [13], [15]. Heterogenity in the paralogous *tpr* gene families of these organisms have been characterized [16], [17], and may be responsible in part for the pathogenic differences and incomplete immunologic cross-protection observed.

Differences in host specificity and in clinical manifestations of these diseases reflect the primary genetic differences between *T. paraluiscuniculi* and *T. pallidum*. In this communication, we report a complete genome sequence of *T. paraluiscuniculi*, strain Cuniculi A and compare this sequence to the published genomes of *T. pallidum* ssp. *pallidum* Nichols, SS14 and Chicago [18]–[20].

## Results

### *T. paraluiscuniculi* Cuniculi A genomic parameters and annotation

The summarized genomic features of *T. paraluiscuniculi* strain Cuniculi A are shown in the Table 1. The genome size of *T. paraluiscuniculi* Cuniculi A (1,133,390 bp) is 4.6, 5.9 and 6.1 kb smaller than the genome size of the previously published *T. pallidum* ssp. *pallidum* Nichols (1,138,011 bp) [18], Chicago (1,139,281 bp) [20] and SS14 genomes (1,139,457 bp) [19], respectively. Similar whole genome nucleotide diversity (π ± SD) of 0.01028±0.00514, 0.01021±0.00511, and 0.01016±0.00508, was revealed by DnaSP v5 software between Cuniculi A genome and Nichols, Chicago, and SS14 genomes, respectively. The deletions in the Cuniculi A genome were not evenly distributed in the genome and were predominantly localized in *tpr* loci and the vicinity of these regions [2], [16]. The overall gene order in the Cuniculi A was identical to both Nichols and SS14 genomes. Out of 1,133,390 bp of the Cuniculi genome, 1,092,714 bp were aligned with the Nichols genome and this part of the genome contained 8074 single nucleotide replacements and additional 1124 bp changes in 224 individual indels. This corresponds to 99.16% sequence identity between the conserved regions of the Nichols and Cuniculi A genomes. Out of 1016 annotated genes encoding proteins in the Cuniculi A genome, gene function was predicted for 650 genes (64%). In addition to genes present in the Nichols genome, 57 hypothetical genes were newly annotated in the Cuniculi A genome. These genes were annotated in the orthologous sequences and did not represent a new genetic material specific to Cuniculi A. Instead, they represented differences in the recent annotation algorithms compared to the previously used ones [18]. The average gene length of these 57 genes was of 234 nt (median length of 162 nt) indicating markedly shorter gene length compared to average and median gene length of all Cuniculi A genes (1006 and 873 bp, respectively). Seventeen automated gene predictions were omitted in the final annotation mainly due to overlap with other already annotated genes. 75 and 280 genes were annotated shorter or longer, respectively, in the Cuniculi A annotation when compared to the length of orthologous genes in the Nichols and SS14 genome annotations. 47 genes originally annotated in the Nichols genome [18] were not annotated in the Cuniculi A genome as a result of differences in gene prediction criteria. For all of these 47 genes, orthologous sequences were found in the Cuniculi A genome. All of these 47 genes encoded short hypothetical proteins with average gene length of 196.4 bp (median of 150 bp).

**Table 1:** Summary of the genomic features of *T. paraluiscuniculi* strain Cuniculi A.

Genome parameter	Value
Genome size	1,133,390 bp
G+C content	52.8 %
No. of predicted genes	1070 including 54 untranslated genes
Intergenic region length	62,494 bp (5.5% of the genome length)
Average/median gene length	1006/873
No. of genes encoded on plus/minus DNA strand	577/493
No. of genes encoding hypothetical proteins similar to proteins of known function	650
No. of genes encoding conserved hypothetical proteins	139
No. of genes encoding hypothetical proteins	227
No. of pseudogenes or gene fragments	51 (21 in genes with predicted function and 30 in hypothetical and predicted genes)
No. of fused genes	52 (resulting in 25 corresponding genes in Cuniculi A, Table S1)
No. of tRNA loci	45
No. of rRNA operons	2 (6 genes)
No. of other stable RNAs	3

Gene fragments and fused genes were identified in comparison to the genome sequence of *Treponema pallidum* subspecies *pallidum* Nichols.

Alignment of the Cuniculi A genome with the annotated Nichols genes identified 84 orthologous Cuniculi A regions/genes containing internal frameshifts and/or major sequence changes. In the Cuniculi A genome, 63 genes (6.2% of 1016 protein-encoding genes) were annotated in these orthologous loci. Genes with major sequence changes were defined as those causing more than 10 continuous amino acid replacements (or indels) in the corresponding protein sequence or showing more than 20 dispersed amino acid changes in the Cuniculi A protein compared to the Nichols ortholog. Changes in protein length at the N-terminus resulting from predictions of longer Cuniculi A genes were not considered as major sequence changes if there was an existing potential downstream start codon at corresponding gene position as in the Nichols genome. A set of 134 (13.2% of 1016 protein-encoding genes) predicted Cuniculi A genes encoded identical proteins to predicted Nichols proteins and 819 genes (80.6%) encode proteins with one or several amino acid replacements.

### Gene fusions

Compared to the published Nichols genome, 52 Nichols orthologs were fused into 25 genes in the Cuniculi A genome (see Table S1). In two cases, three genes annotated in the Nichols genome were fused to one gene in the Cuniculi A genome (TP0006, TP0007, TP0008 and TP0174, TP0175, TP0176). Similar situation was also found in the genome of *T. pallidum* subspecies *pertenue* Samoa D where sequencing revealed fusion of 48 Nichols orthologs into 23 genes (data not shown). An ongoing resequencing of the Nichols genome (P. Pospíšilová, personal communication) revealed that most of the observed gene fusions are also present in the Nichols genome indicating that the published Nichols sequence [18] contains dozens of sequencing errors. To test whether these Nichols changes are sequencing errors or intrastrain adaptive mutations, we analyzed 208 nucleotide positions in which the resequenced Nichols genome differs from the published Nichols version [18] in 3 *T. pallidum* strains including Chicago [20] and preliminary DAL-1 and Mexico A whole genome sequences (unpublished data). Out of 208 nucleotide differences found in the resequenced Nichols genome, 179 (86.1%) were present in all 3 tested genomes, 12 (5.8%) were present in one or two other genomes and 17 (8.2%) were specific for the newly sequenced Nichols strain. The presence of majority of nucleotide changes (identified in the resequenced Nichols genome) in three other *T. pallidum* genomes indicates sequencing errors in the published Nichols genome [18] rather than recently emerged intrastrain adaptive mutations.

### Whole genome fingerprinting and sequencing error rate

Whole genome fingerprinting was used to assess the overall genome assembly of the Cuniculi A genome. Fingerprinting of the Cuniculi A genome described earlier [2] was extended by additional restriction enzyme analyses to reduce the length of the resulting DNA fragments. The *in silico* restriction mapping was compared to experimentally obtained restriction digest patterns. In the final assembly, 2017 restriction target sites were experimentally verified resulting in average length of assessed DNA fragment of 562 bp. The 2017 restriction target sites corresponded to a total sequence length of 11,702 bp (1.0% of the genome length). Since no discrepancies between *in silico* and experimental restriction analysis were observed, the expected corresponding sequencing error rate in the Cuniculi A genome was set to the order of 10^−4^ or better.

### *T. paraluiscuniculi* genes encoding identical proteins as corresponding orthologs in the Nichols genome

Altogether, 134 (13.2%) of Cuniculi A genes were found to encode identical proteins to those encoded in the Nichols genome (Table S2 and S3). 35 of these genes (Table S3) encoded proteins of unknown function and the remaining 99 genes encoded proteins involved in the translation of mRNA (32 genes), general metabolism (24 genes), transport (12 genes), flagellar synthesis (11 genes), gene regulation (6 genes) and other functions (14 genes). Conservation of these proteins and their predominant involvement in translation and general metabolism indicate that these genes are housekeeping genes under strong negative selection in the genus *Treponema*. The identical genes encoding proteins of unknown function (35, or 26.1%) may encode proteins needed for as yet unidentified essential functions, including general metabolic processes. Although the median RNA transcript level of these 35 genes in *T. pallidum* ssp. *pallidum* Nichols [21] is lower than that of the 99 identical genes with annotated functions (0.92 and 1.37, respectively), it is close to the median value for all genes with annotated functions (0.95).

### Genes containing frameshifts and/or major sequence changes (MSC) in *T. paraluiscuniculi* functional gene groups

*T. paraluiscuniculi* genes were classified into 7 functional groups according to the modified classification used by Fraser et al. [18] (Table 2). A set of 161 genes (9.8% of all protein-encoding genes) encoding proteins involved in general metabolic functions was used as an internal standard for comparisons with genes from other functional groups. The number of genes containing frameshifts and/or major sequence changes (related to Nichols orthologs) was compared to the number of these genes present in the general metabolism group (1.9%, Table 2) and statistically significant differences were found in the group of virulence factors (41.9%, p<0.001), in genes with an unknown function (14.0%, p<0.001), and in genes involved in DNA metabolism (7.8%, p = 0.037).

**Table 2:** Genes containing frameshifts and/or major sequence changes* (MSC) in *T. paraluiscuniculi* functional gene groups.

Functional gene group	No. of genes containing frameshifts and/or MSC^a^ (%)	Total no. of genes	Statistical significance
General metabolism	3 (1.9)	161	
Cell processes; cell structure	6 (4.8)	124	ns^b^
DNA replication, repair, recombination	4 (7.8)	51	0.037
Regulation; transcription; translation	4 (2.3)	172	ns
Transport	2 (1.8)	112	ns
Virulence; potential virulence factors	13 (41.9)	31	p<0.001
Unknown	52 (14.0)	372	p<0.001

*Major sequence changes were defined as continuous amino acid replacements comprising 10 and more residues or 20 and more dispersed amino acid replacements. Annotations of the Cuniculi A genes predicting longer proteins at the N-terminus with existing potential downstream start codons at corresponding positions as in the Nichols genome were not considered as major sequence changes.

a when compared to Nichols orthologs.

b ns, not statistically significant.

### Hypothetical genes with internal frameshifts and/or major sequence changes in the *T. paraluiscuniculi* genome

Altogether, 52 Cuniculi A hypothetical genes corresponding to 54 Nichols orthologs (Table S4) in the *T. paraluiscuniculi* genome showed frameshifts (21 genes), partial or complete gene deletions (7 genes), internal stop codons (2 genes) or multiple nucleotide changes (22 genes). Twelve of these proteins were predicted inner or outer membrane proteins and 9 of them were identified as antigens [22]. Three of these 52 proteins (TP0133, TP0462, and TP0895) were predicted lipoproteins [23], and these 3 proteins were also identified as antigens. These results indicate that the *T. paraluiscuniculi* genome may be undergoing gene degradation and loss relative to *T. pallidum* subsp. *pallidum*, consistent with a decreased requirement for these genes in the rabbit tissue niche inhabited by *T. paraluiscuniculi*. Altogether, for 25 out these 52 Cuniculi A hypothetical genes, the Ka/Ks ratios and predicted type of selection were calculated (Table S4). Most of the genes (20) were found under neutral selection, while 4 genes under purifying and one under positive selection.

### Cuniculi A genes with predicted cell function containing internal frameshifts and/or major sequence changes

**Figure 1:**
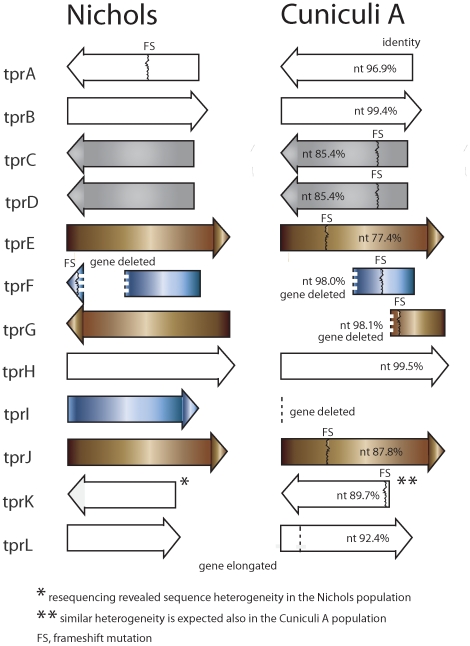
A schematic representation of *tpr* genes in the Cuniculi A and Nichols genomes. Identities at nucleotide levels of Cuniculi A and Nichols genomes are shown. Colors indicate sequence similarities among paralogous *tpr* genes, i.e. sequence similarities within the *T. paraluiscuniculi* genome (e.g. *tprC* and *tprD* genes are identical). In the Cuniculi A genome, reverted frameshift mutation (in *tprA*), frameshift mutations (in *tprC,D,E,F,G,J,K*), deletions (in *tprF,G,I*) and gene elongation are present (in *tprL*). The *tprF* deletion shown in the Nichols genome is based on the *tprF* sequence taken from *T. pallidum* ssp. *pertenue* Samoa D genome (data not shown). In the Cuniculi A and Nichols *tprK* genes, shorter gene versions (starting with the next available downstream start codon) are expected rather than the presence of frameshift mutation in the Cuniculi A *tprK*.

Altogether, 32 Cuniculi A genes with defined or predicted functions were found to contain frameshifts or major deletions (resulting in 21 pseudogenes), major sequence changes (8 genes), or reverted frameshift mutations (3 genes) (Table 3). Ten out of these 32 genes were *tpr* genes encoding paralogous proteins with sequence similarity to the major surface protein (Msp) of *Treponema denticola* [24]. A schematic representation of all *tpr* genes in the Cuniculi A genome is shown in Fig. 1. In addition to these potential virulence factors, another three proteins encoded by TPCCA_0136 (fibronectin-binding protein), TPCCA_0326 (tp92, outer membrane protein), and TPCCA_0433 (acidic repeat protein, Arp protein) showed indels and major sequence changes. Together with TPCCA_0760 (penicillin-binding protein), these proteins are important treponemal antigens and/or cell envelope structures [25]–[27]. Another gene potentially involved in cell wall biosynthesis, the Cuniculi A polysaccharide biosynthesis *capD* gene (TPCCA_0077), contained an internal stop codon predicted to result in gene inactivation. The Cuniculi A transmembrane chemoreceptors (Mcp proteins) either showed major sequence changes (TPCCA_0040 and TPCCA_0488, Table S4) or showed a relatively high number of amino acid replacements (TPCCA_0639, TPCCA_0640; 14 and 10 aa changes, respectively; data not shown).

**Table 3:** *T. paraluiscuniculi* Cuniculi A genes with predicted cell function containing internal frameshifts and/or MSC* compared to the Nichols orthologs.

Gene	Gene name	Gene/protein function	Type of change	Functional group/cell function	Ka/Ks ratio (if applicable) and estimation of selection type^a^	Remark/reference
TPCCA_0009	***tprA***	Tpr protein	reverted frameshift mutation, MSC	potential virulence factors		[16] authentic frameshift mutation in the Nichols genome
TPCCA_0117	***tprC***	Tpr protein	frameshift mutation			
TPCCA_0131	***tprD***	Tpr protein	frameshift mutation			[2], [16]
TPCCA_0313	***tprE***	Tpr protein	frameshift mutation			[2], [16]
TPCCA_0316	***tprF***	Tpr protein	partial gene deletion		1.19, neutral selection	[2], [16]
TPCCA_0317	***tprG***	Tpr protein	frameshift mutation, partial gene deletion			[2], [16]
TPCCA_0620	***tprI***	Tpr protein	gene deleted			[2], [16]
TPCCA_0621	***tprJ***	Tpr protein	frameshift mutation			[2], [16]
TPCCA_0897	***tprK***	Tpr protein	frameshift mutation, MSC			[16]
TPCCA_1031	***tprL***	Tpr protein	MSC		1.64, positive selection	[2], [16]
TPCCA_0136^b^		fibronectin binding protein	MSC	virulence	1.05, neutral selection	lipoprotein, [26]
TPCCA_0326^b^	***tp92***	outer membrane protein	MSC, gene deletions^c^		0.57, purifying selection	
TPCCA_0433^b^ *^,^* d	***arp***	treponemal conserved hypothetical protein	MSC, gene insertions		7.37, positive selection	[2]
TPCCA_0077	***capD***	capsular polysaccharide biosynthesis protein	internal stop codon	cell structure	0.17, neutral selection	
TPCCA_0040	***mcp1***	probable methyl-accepting chemotaxis protein	frameshift, MSC	cell processes		
TPCCA_0488	***mcp2***	methyl-accepting chemotaxis protein	MSC		1.45, neutral selection	
TPCCA_0760	***pbp2***	penicillin-binding protein	internal stop codon		0.033, purifying selection	
TPCCA_0801	***clpA2***	S14 family endopeptidase ClpA	frameshift mutation			
TPCCA_0936		probable hemolysin	internal stop codon		0.33, neutral selection	
TPCCA_0220^b^		anti-sigma factor antagonist	frameshift mutation	regulation		
TPCCA_0461		probable transcriptional regulator	frameshift mutation			
TPCCA_0511		CarD family transcriptional regulator	frameshift mutation			
TPCCA_0520		sensor histidine kinase	reverted frameshift mutation			authentic frameshift mutation in the Nichols genome
TPCCA_0103	***recQ***	ATP-dependent helicase	frameshift mutation	DNA replication, repair, recombination		
TPCCA_0310	***ssb2***	probable single-stranded DNA-binding protein	frameshift mutation			[2]
TPCCA_0898	***recB***	exodeoxyribonuclease V beta subunit	MSC		0.48, purifying selection	
TPCCA_1023	***recX***	recombination regulator RecX	frameshift - missing stop codon			
TPCCA_0735	***gltD***	glutamate synthase (NADPH)	frameshift mutation	general metabolism		
TPCCA_0812	***fadD2***	probable long-chain-fatty-acid–CoA ligase	reverted frameshift mutation			authentic frameshift mutation in the Nichols genome
TPCCA_0104	***ushA***	bifunctional 5′-nucleotidase/UDP-sugar diphosphatase	frameshift mutation			lipoprotein
TPCCA_0309		probable polar amino acid ABC superfamily ATP binding cassette transporter, binding protein	frameshift mutation	transport		[2]
TPCCA_0545	***mglB***	sugar ABC superfamily ATP binding cassette transporter, binding protein	partial deletion at 5′end – start codon missing		0.47, neutral selection	

*Major sequence changes are defined in Table 2.

a Ka/Ks ratios were calculated by the MEGA4 software [52] and the selection test was calculated using the Kumar model [51].

b corresponding protein identified as antigen [22].

c deleted/changed serine rich regions mediates in some proteins attachment to cell surface [25].

d fused genes (see Table S1).

Several Cuniculi A genes with frameshifts belonged to the family of genes with predicted regulatory functions including TPCCA_0220 (anti-sigma factor antagonist), TPCCA_0461 (probable transcriptional regulator), TPCCA_0511 (CarD family transcriptional regulator), and TPCCA_0520 (sensor histidine kinase). In contrast to TP0520 encoded in the genome of *T. pallidum* ssp. *pallidum* (strains Nichols and SS14) and in the *T. pallidum* ssp. *pertenue* (strains Samoa D, CDC-2, Gauthier; M. Zobaníková, D. Čejková, personal communication), TPCCA_0520 of *T*. *paraluiscuniculi* encodes full length sensor histidine kinase suggesting possible role of this protein in gene regulation. In addition to the previously mentioned *tprA* and TPCCA_0520 genes, reversion of frameshift mutation in TPCCA_0812 (*fadD*, probable long-chain-fatty-acid–CoA ligase) was also found in the Cuniculi A genome.

Four genes encoding proteins involved in DNA processing (RecB, RecQ, RecX, and Ssb) also had altered open reading frames in the Cuniculi A strain relative to the Nichols strain. The frameshift mutation in the *recQ* gene resulted in premature truncation of RecQ, whereas RecX and Ssb proteins were elongated at C-termini. RecB showed multiple amino acid changes. Other genes with major sequence changes involved *gltD* (TPCCA_0735, glutamate and proline biosynthesis), *ushA* (TPCCA_0104, bifunctional 5′-nucleotidase/UDP-sugar diphosphatase), TPCCA_0309 (probable polar amino acid ABC transporter), and *mglB* (TPCCA_0545, sugar ABC transporter).

For 11 out of these 32 Cuniculi A genes, selection type was predicted (Table 3). While majority of genes were under neutral (6) or purifying selection (3), two genes including *tprL* and *arp* were found under positive selection.

Taken together, genes encoding several potential virulence determinants, including Tpr proteins, other membrane proteins, cell wall biosynthesis proteins, gene regulatory proteins and components of the DNA repair process, are significantly altered in the *T. paraluiscuniculi* Cuniculi A genome relative to the *T. pallidum* genomes sequenced to date.

## Discussion

The complete genome sequence of *T. paraluiscuniculi* Cuniculi A was determined by combining the data obtained by Illumina and Sanger sequencing and microarray hybridization approaches. The 454 sequencing data were used as a scaffold for the assembly and the final genome sequence was verified by genomic fingerprinting. This analysis of the Cuniculi A genome revealed striking similarity with other sequenced treponemal genomes (99.16% sequence identity between the conserved regions of the Nichols and Cuniculi A genomes), including identical gene orders, despite the differences in the host specificity and clinical manifestations of infections caused by *T. paraluiscuniculi* and *T. pallidum* ssp. *pallidum*. The Cuniculi A genome size (1,133,390 bp) is about 4.6 and 6.1 kb smaller than the genome size of the previously published *T. p.* ssp. *pallidum* Nichols and SS14 genomes, respectively. Additionally, an insertion harboring a *tprK*-like sequence (1.3 kb) in the intergenic region between TP0126 and TP0127 is present in a subpopulation of the Nichols strain [28] resulting in 5.9 kb difference between Cuniculi A and Nichols genomes. In unpublished studies, the genome of *T. pallidum* subspecies *pertenue* Samoa D (1,139,330 bp) was also found to be ∼6.0 kb larger than the Cuniculi A genome (D. Čejková, personal communication). As shown by Strouhal et al. [2], the smaller Cuniculi A genome is a result of deletions localized mainly around *tpr* genes. In addition to decreased genome size, the Cuniculi A genome contained markedly higher number of pseudogenes and gene fragments. In the Nichols genome, only 9 genes containing authentic frameshifts were identified [18]. Besides these 9 genes with authentic frameshifts there are additional 6 pseudogenes in the SS14 genome (5 genes with frameshifts and one with nonsense mutation) [19]. In the genome of Samoa D, there are 13 pseudogenes (M. Zobaníková, personal communication). The number of pseudogenes and gene fragments (51) in the Cuniculi A genome thus markedly exceeds the number in Nichols, SS14 and Samoa D genomes, respectively.

In the Cuniculi A genome, there are 25 genes representing fusions of 52 Nichols orthologs. In most cases, Nichols orthologs are separated because of sequencing errors in the published whole genome Nichols sequence [18]. Therefore, most of these gene fusions do not represent true differences in the compared genomes. In contrast, missing genes, gene fragments, pseudogenes, and genes encoding proteins with many amino acid changes were considered important differences.

About 13% of the Cuniculi A genes were found to encode identical proteins to those encoded in the Nichols genome, indicating strong conservation of protein sequence in these proteins. Most of these genes encoded housekeeping proteins and likely represent a set of highly conserved treponemal genes important for basic treponemal cellular functions. The 35 genes encoding identical hypothetical proteins in both genomes are also candidates for important cellular functions. Indeed, the functions of three of these proteins were predicted recently: TP0650 as a protein involved in translation, TP0772 as a transcriptional regulator and TP0941 as a regulator of motility [29].

Comparison of number of genes containing frameshifts and/or major sequence changes (when compared to Nichols orthologs) within the Cuniculi A functional gene groups revealed a high percentage of these genes in the group of virulence factors, in genes involved in DNA metabolism, and in a group of genes with unknown function. These findings suggest that the accumulation of changes in genes encoding predicted virulence factors and genes with unknown functions (some of them are potential candidates for virulence factors, see below) is the reason for the loss of *T. paraluiscuniculi* infectivity to humans. Moreover, affected genes involved in DNA replication, repair, and recombination could suggest their possible role in the acceleration of *T. paraluiscuniculi* evolution.

Fifty-two hypothetical genes with internal frameshifts and/or major sequence changes were found in the *T. paraluiscuniculi* genome (Table 2 and S4). The data available from the previously published transcriptome analysis that mapped the Nichols strain gene expression levels during experimental rabbit infection [21] clearly showed that these genes are actively transcribed during infection. Moreover, the median transcript levels of these genes was considerably higher than the median gene expression rate of all genes of unknown function (1.46 versus 0.86) indicating that these genes are likely to represent true genes in the Nichols genome playing an important role during infection. These hypothetical genes are therefore candidates for important virulence factors of *T. pallidum* and should attract interest in future syphilis research. In *T. paraluiscuniculi*, most of the genes (where the type of selection could be calculated) belonging to this group were found under neutral selection suggesting a genetic inactivity of these genes. The one positively selected gene, TP0031, could represent gene involved in adaptation of *T. paraluiscuniculi* to rabbits. In addition, 12 of hypothetical genes with internal frameshifts and/or major sequence changes were predicted to be inner or outer membrane proteins. *T. pallidum* whole–genome antigen screen, which tested 882 gene products [22], identified 106 antigens recognized by rabbit antibodies obtained from infected rabbits. Out of these 106 identified antigens, 9 antigens correspond to the group of 52 Cuniculi A hypothetical protein genes that have frameshifts or major sequence changes. Interestingly, the group of predicted membrane and outer membrane proteins and the group of identified antigens did not overlap, probably as a result of problematic recombinant production of membrane proteins in *E. coli*. In addition, 22 hypothetical proteins were identified to interact with proteins of known function including predominantly cell wall structures and antigens, regulatory and metabolic proteins [29]. Altogether, more than half of these proteins are likely involved in cell wall structure, and several others might be involved in gene regulation.

The most affected group of genes in the Cuniculi A genome was the family of paralogous *tpr* genes. Although the precise role of individual *tpr* genes in the treponemal infection remains unclear, there is an expanding evidence of the role of *tpr* genes in treponemal pathogenicity and host specificity. In the genome of *T. pallidum* subsp. *pallidum* Nichols, there are 12 paralogous *tpr* genes [18]. It was shown that Tpr proteins induced an antibody response during infection, and exhibit heterogeneity both within and between the *T. pallidum* subspecies and strains examined [30]–[32]. Tpr proteins are thought to be involved in pathogenesis and/or immune evasion. A model of gene conversion-driven antigenic variation of TprK during experimental infection was proposed [33]. Differences between *Treponema* species and subspecies in *tpr* gene content and expression are thought to be important determinants of pathogenesis and immunogenicity [16], [17]. Out of 12 *tpr* genes in the Cuniculi A genome (Fig. 1), only four are intact: *tprA*, *tprB*, *tprH* and *tprL*. The remaining 8 genes contain frameshifts and/or deletions. Interestingly, the *tprA* gene in the Nichols genome contains an authentic frameshift resulting in inactive *tprA* gene. This also applies for the SS14 genome [19]. In contrast, *tprA* in the Cuniculi A (and also in the Samoa D genome; D. Čejková, personal communication) did not contain this frameshift mutation and appears to be functional. In addition to a regular copy of *tprK*, *T. pallidum* contains a *tprK*-like sequence localized in the 1.3 kb insertion present in a part of treponemal population [28]. A similar situation also applies for *T. p.* ssp. *pertenue* genomes, where this site also showed intrastrain heterogeneity (data not shown). The region containing the *tprK*-like sequence was not found in the *T. paraluiscuniculi* [2]. The major differences observed in *tpr* genes between *T. paraluiscuniculi* and *T. pallidum* suggest their role in the host range specificity. In fact, only *tprA,B,H,L* genes appear to be functional in the Cuniculi A genome. In contrast, *tprC,D,E,F,G,I,J,K* were affected in the Cuniculi A genome. The *tprE,G,J* genes were shown to be variably expressed in individual *T. pallidum* clinical isolates with guanosine homopolymers in promoter regions modulating their gene expression [34]. Interestingly, six *tpr* genes including *tprC,D,E,F,I,J* out of 8 affected were recently predicted as genes encoding rare outer membrane proteins (OMPs) in *T. pallidum* [35]. These findings suggest the role of Tpr rare OMPs in *T. pallidum* infectivity to humans. Out of 4 functional *tprA,B,H,L* genes, the *tprB,L* were predicted to encode rare OMPs (*T. paraluiscuniculi* TprL had a predicted signal sequence), suggesting that OMPs may be important also during *T. paraluiscuniculi* infection of rabbits.

There appear to be important differences between *T. paraluiscuniculi* Cuniculi A and other treponemal strains with regard to DNA recombination genes. In the Cuniculi A strain, the mutation in *recQ* resulted in a predicted RecQ protein without C-terminal, HDCR domain [36]. Although the precise role of this domain remains unclear, the C-terminal RecQ domain binds DNA [37]. The predicted sequence of RecB (important in the RecBCD pathway) has a high number of amino acid changes, and the predicted RecX and Ssp proteins have extended C-terminal sequences. There are also differences in the location of *recX* in Cuniculi A relative to other bacterial species and treponemal strains. In *E. coli*, the RecX protein was shown to inhibit some RecA-mediated functions [38]. In contrast, in an exclusive human pathogen, *N. gonorrhoeae*, RecX enhances RecA activity [39]. Similarly to situation in *N. gonorrhoeae* genome, *recX* in Cuniculi A is not located downstream of (and overlapping with) *recA* but is in a different genome locus; this difference indicates that RecX expression and function may be more similar to those in *N. gonorrhoeae*. Taken together, genetic changes in the *recB*, *recQ* and *recX* genes are consistent with observed increased genetic diversity in the Cuniculi A genome due to an ineffective/modified DNA repair and homologous recombination pathways. Homologous recombination as a major DNA repair process occurs frequently in the bacterial genomes and results in gene conversion. Gene conversion is an important mechanism of evolution of paralogous genes [40], and may be affected in the Cuniculi A genome.

We also examined other genetic differences to assess their potential role in pathogenesis patterns in *T. paraluiscuniculi*. In contrast to TPASS_0520 in *T. p.* ssp. *pallidum* and several other treponemes, TPCCA_0520 of *T*. *paraluiscuniculi* encodes full length sensor histidine kinase suggesting possible role of this protein in gene regulation. *tpr*A and TPCCA_0520 genes are two out of four examples of intact genes present in the Cuniculi A genome but containing frameshift mutations in the ssp. *pallidum*. Several Cuniculi A membrane chemoreceptor proteins (Mcp's) contained major sequence changes (TPCCA_0040, TPCCA_0488, TPCCA_0639, TPCCA_0640) when compared to their Nichols counterparts. Chemotactic proteins thus seem to be one of the most divergent proteins in the Cuniculi A genome, which may correlate with altered chemotaxis signaling patterns.

Several bacterial envelope components contained major sequence changes. Arp protein is of unknown function but it contains repeated predicted fibronectin-binding immunogenic domains [27]. Moreover, diverse repeats were shown to be associated with sexual transmission route of treponemal pathogens [41]. Interestingly, the *arp* gene was found under a strong positive selection in the Cuniculi A genome, supporting its potential role in rabbit infection. TPCCA_0136, fibronectin- and laminin-binding protein is an outer membrane protein showing both inter- and intrasubspecies variable sequences. Immunization with recombinant protein delayed ulceration but did not prevent infection or the formation of lesions [26]. The immunization with recombinant Tp92 partially protected rabbits from subsequent *T. pallidum* challenge [25]. Sequence changes in genes encoding important antigens are one of the most probable reasons for changes in pathogenicity and host specificity. In *Rickettsia prowazekii*, the *capD* gene codes for an epimerase involved in capsular polysaccharide biosynthesis. The Cuniculi A *capD* was inactive. Since exopolysaccharides are important bacterial virulence factors, *capD* mutation in the Cuniculi A may be one of the reasons for decreased virulence of this strain. Beside these genes, altered genes with predicted regulatory functions (TPCCA_0220, TPCCA_0461, and TPCCA_0511) suggested differences in the gene regulatory network in the Cuniculi A genome. Changed regulatory network in the Cuniculi A genome and the resulting down- or upregulation of individual genes could be added to potential reasons for observed decreased virulence of Cuniculi A strain.

Taken together, the decreased size of the genome, marked increase in number of pseudogenes, affected genes involved in cell envelope biosynthesis and structure and multiple genetic changes in the proteins involved in DNA recombination, cell signaling and gene regulation appear to be the major reasons for narrower host specificity. Downsizing of the genome and accumulation of pseudogenes is common for bacteria adapting to simpler host-associated niches [42]. The loss of infectivity of *T. paraluiscuniculi* to humans may represent such a process. On the other hand, adaptation of *T. paraluiscuniculi* to rabbits resulting in more efficient infection of this host could be a result of additional changes that may include positively selected *tprL*, *arp*, and TP0031 genes and/or a number of dispersed mutations throughout the *T. paraluiscuniculi* genome. *T. paraluiscuniculi* thus appears to be treponeme in the process of adaptation to a single host (rabbit) and therefore is likely to be a descendant of *pallidum*- or *pertenue*-like ancestors rather than the opposite.

## Materials and Methods

### Isolation of *T. paraluiscuniculi* chromosomal DNA

*T. paraluiscuniculi* strain Cuniculi A was initially isolated from an infected rabbit by Drs. Paul Hardy and Ellen Nell; the strain was kindly provided by Dr. Sheila A. Lukehart at the University of Washington. Organisms were propagated by intratesticular inoculation of rabbits, extracted, and purified by Hypaque gradient centrifugation as described previously [18], [43]. Genomic DNA was prepared according to the protocol published earlier [18].

### DNA sequencing

The Cuniculi A genomic DNA (2.1 µg) was used for sequencing-by-synthesis (based on pyrosequencing) using GS20 sequencing machine (454 Life Sciences Corporation, Branford, CT, USA). Sequencing resulted in 398 individual trimmed contigs with a total contig size of 1,133,704 bp (average contig length of 2848 bp, contig length ranging from 102 to 22,217 bp). A subset of 330 individual contigs showed hits to published Nichols treponemal DNA [18], covering 1,128,602 bp and leaving 4,788 bp unsequenced. The number of individual reads in these 330 contigs was 204,765 representing total read length of 20,443,023 bp. The corresponding sequencing coverage for the Cuniculi A genome was 18.04. Most of contings not related to treponemes showed similarity to rabbit sequences, probably as a result of contamination of Cuniculi A DNA by rabbit DNA during preparation of chromosomal DNA.

Parallel to pyrosequencing, an Illumina (Illumina, San Diego, CA, USA) sequencing approach was employed using the Genome Analyzer sequencing machine. Illumina reads (36 bp each) were assembled into 726 contigs using Velvet short read assembler [44]. Total number of reads (3,053,564) represented total read length of 109,928,304 bp (97x coverage). Some of the 726 contigs overlap by few bp and therefore the number of gaps dropped to the number 475, representing total gap length of 33,634 bp.

Sanger sequencing of Cuniculi A sequences was used to assess the quality of DNA sequencing and to finish the whole genome sequence. Approximately 150 PCR products, generated with primers used for other treponemal genome projects were sequenced to provide a comparison to the Illumina- and 454-generated sequences. All 50 discrepancies were in the 454 sequence results, and included 43 false insertions, 3 false deletions and 4 substitutions. Twenty-four out of 46 indels were found in homopolymeric regions). Based on these results, we considered the Illumina sequences to be more accurate and utilized them for generation of the complete genome sequence.

An additional approach, the CGS strategy [19] was used for determination of the Cuniculi A genome sequence. Oligonucleotide arrays of 29-mers derived from the Nichols sequence, covering both strands, were hybridized separately to fluorescently-labeled Nichols and Cuniculi A genomic DNA. Equal hybridization signals in both preparations of genomic DNA indicated identical sequences, whereas decreased hybridization with the Cuniculi A DNA occurred in regions with sequence differences or indels. This information was used to help ‘fill in’ the sequence gaps between Illumina contigs. All discrepancies in gap regions were resolved by traditional Sanger sequencing.

### Whole genome fingerprinting

Whole genome fingerprinting [45] results for the Cuniculi A genome [2] were used for verification of the genome assembly. Briefly, primers designed for *T. pallidum* subsp. *pallidum* and template DNA from *T. paraluiscuniculi* Cuniculi A were used to produce large, 5 to 28 kb amplicons spanning the entire genome; these were then digested with multiple enzymes to provide a macro restriction map. The Cuniculi A genomic sequence was used for simulated restriction digest *in silico* and these data were compared with experimentally obtained data. Altogether, 19 individual restriction enzymes were used including *Acc* I (194 verified restriction target sites), *Asc* I (2), *Bam*H I (222), *Cla* I (107), *Eco*R I (157), *Eco*R V (200), *Hin*d III (258), *Kpn* I (112), *Mlu* I (277), *Mse* I (8), *Nco* I (61), *Nde* I (1), *Nhe* I (14), *Rsr* II (20), *Sac* I (86), *Spe* I (25), *Sph* I (13), *Xba* I (68) or *Xho* I (191) enzymes (NEB) either alone or in combinations. Three enzymes, *Bam*H I, *Eco*R I and *Hin*d III, were used for restriction analysis of all amplicons. The use of other enzymes was optional depending on length of restriction fragments and the availability of restriction target sites. To ascertain the experimental error of WGF, the lengths of 250 individual DNA fragments in 5 fragment intervals (50 fragments per interval) including 0.2–0.5 kb, 0.5–1 kb, 1–2 kb, 2–3 kb, and 3–4 kb, respectively, were measured from agarose gels by AlphaView Software Version 3.0 (Alpha Innotech, San Leandro, CA) and calculated from *in silico* data. The average error for each interval represented an average difference between experimental and calculated fragments sizes. The average errors were calculated to 10.9 bp, 16.8 bp, 22.3 bp, 38.9 bp and 52.5 bp in 0.2–0.5 kb, 0.5–1 kb, 1–2 kb, 2–3 kb and 3–4 kb intervals, respectively. A set of the smallest available 722 fragments (covering the entire Cuniculi A genome), with length ranging between 0.2 and 4.0 kb (average length of 1712 bp) and covering slightly more than the length of Cuniculi A genome due to overlaps of amplified regions (1,235,806 bp), were selected and the error rate was calculated based on number of DNA fragments in each individual size interval. The average error for all analyzed DNA restriction fragments was calculated to 27.9 bp (1.6% of average fragment length) with the variation range between 0 and 132 bp.

#### Gene prediction and annotation

Gene prediction and annotation was performed according to the automated annotation scheme used at The Genome Center at Washington University [46]. Genes (TPCCA genes) were predicted by the Glimmer and GeneMark programs [47], [48]. The automated annotation was modified by comparison with the published genome sequences of the *T. pallidum* Nichols and SS14 genomes [18], [19]. Genome alignments were performed using the *Consed* finishing tool [49].

### DNA sequence analyses and statistical analyses

DNA sequence analyses were performed using the *DnaSP* software, version 5.10 [50]. Whole genome nucleotide diversity (π) between Cuniculi A and individual sequenced *T. pallidum* genomes including Nichols, Chicago and SS14 strains was calculated, respectively. The number of synonymous substitutions per a synonymous site (Ks), the number of nonsynonymous substitutions per a nonsynonymous site (Ka), the Ka/Ks ratios, and the codon-based test for estimation of selection type were calculated using the Kumar model [51] and the MEGA4 software [52]. Statistical significance of the number of genes containing frameshifts and/or major sequence changes (MSC) in *T. paraluiscuniculi* functional gene groups was calculated using standard methods derived from the binomial distribution, including the two-tailed test. For statistical calculations, *STATISTICA* program, version 8.0, (StatSoft, Tulsa, OK, USA) was used.

### Nucleotide sequence accession numbers

The nucleotide sequences reported in this study were deposited in the GenBank under the accession number CP002103.

## Supporting Information

Table S1 **Genes fused in the** ***T. paraluiscuniculi*** **Cuniculi A genome when compared to the previously annotated** ***T. pallidum*** **subsp.** ***pallidum*** **Nichols genome** [**18**] **.**
(DOC)

Table S2 **99 genes encoding identical proteins in** ***T. paraluiscuniculi*** **Cuniculi A and** ***T. pallidum*** **subsp.** ***pallidum*** **Nichols genomes.**
(DOC)

Table S3 **35 genes of unknown function encoding identical proteins in** ***T. paraluiscuniculi*** **Cuniculi A and** ***T. pallidum*** **subsp.** ***pallidum*** **Nichols genomes.**
(DOC)

Table S4 ***T. paraluiscuniculi*** **Cuniculi A genes with unknown cell function containing internal frameshifts and/or major sequence changes (MSC) compared to the Nichols orthologs.**
(DOC)

## References

[1] Graves S, Downes J (1981). Experimental infection of man with rabbit-virulent *Treponema paraluis-cuniculi*.. Br J Vener Dis.

[2] Strouhal M, Šmajs D, Matějková P, Sodergren E, Amin AG (2007). Genome differences between *Treponema pallidum* subsp *pallidum* strain Nichols and *T. paraluiscuniculi* strain Cuniculi A.. Infect Immun.

[3] Jacobsthal E (1920). Untersuchungen über eine syphilisähnliche Spontanerkrankung des Kaninchens (Paralues-cuniculi).. Derm Wschr.

[4] Smith JL, Pesetsky BR (1967). The current status of *Treponema cuniculi*: Review of the literature.. Br J Vener Dis.

[5] DiGiacomo RF, Talburt CD, Lukehart SA, Baker-Zander SA, Condon J (1983). *Treponema paraluis-cuniculi* infection in a commercial rabbitry: epidemiology and serodiagnosis.. Lab Anim Sci.

[6] DiGiacomo RF, Lukehart SA, Talburt CD, Baker-Zander SA, Condon J (1984). Clinical course and treatment of venereal spirochaetosis in New Zealand white rabbits.. Br J Vener Dis.

[7] Schell RF, Azadegan AA, Nitskansky SG, Lefrock JL (1982). Acquired resistance of hamsters to challenge with homologous and heterologous virulent treponemes.. Infect Immun.

[8] Turner TB, Hollander DH (1957). Biology of the treponematoses..

[9] Baker-Zander SA, Lukehart SA (1984). Antigenic cross-reactivity between *Treponema pallidum* and other pathogenic members of the family *Spirochaetaceae*.. Infect Immun.

[10] Hougen KH, Birch-Andersen A, Jensen HJ (1973). Electron microscopy of *Treponema cuniculi*.. Acta Pathol Microbiol Scand Microbiol Immunol.

[11] Norris SJ, Pope V, Johnson RE, Larsen SA, Murray PR, Baron EJ, Pfaller MA, Jorgensen JH, Yolken RH (2003). Treponema and other human host-associated spirochetes.. Manual of Clinical Microbiology.

[12] Peeling RW, Hook EW (2006). The pathogenesis of syphilis: the Great Mimicker, revisited.. J Pathol.

[13] Digiacomo RF, Lukehart SA, Talburt CD, Baker-Zander SA, Giddens WE (1985). Chronicity of infection with *Treponema paraluis-cuniculi* in New Zealand white rabbits.. Genitourin Med.

[14] Levaditi C, Marie A, Nicolau S (1921). Virulence pour l'homme du spirochète de la spirillose spontanée du lapin.. C R Acad Sci.

[15] Khan AS, Nelson RAJ, Turner TB (1951). Immunological relationships among species and strains of virulent treponemes as determined with the treponemal immobilization test.. Am J Hyg.

[16] Giacani L, Sun ES, Hevner K, Molini BJ, Van Voorhis WC (2004). Tpr homologs in *Treponema paraluiscuniculi* Cuniculi A strain.. Infect Immun.

[17] Gray RR, Mulligan CJ, Molini BJ, Sun ES, Giacani L (2006). Molecular evolution of the *tprC*, *D*, *I*, *K*, *G*, and *J* genes in the pathogenic genus *Treponema*.. Mol Biol and Evol.

[18] Fraser CM, Norris SJ, Weinstock GM, White O, Sutton GG (1998). Complete genome sequence of *Treponema pallidum*, the syphilis spirochete.. Science.

[19] Matějková P, Strouhal M, Šmajs D, Norris SJ, Palzkill T (2008). Complete genome sequence of *Treponema pallidum* ssp *pallidum* strain SS14 determined with oligonucleotide arrays.. BMC Microbiol.

[20] Giacani L, Jeffrey BM, Molini BJ, Le HT, Lukehart SA, et al (2010). Complete genome sequence and annotation of the *Treponema pallidum* subsp. *pallidum* Chicago strain.. J Bacteriol.

[21] Šmajs D, McKevitt M, Howell JK, Norris SJ, Cai WW (2005). Transcriptome of *Treponema pallidum*: Gene expression profile during experimental rabbit infection.. J Bacteriol.

[22] McKevitt M, Brinkman MB, McLoughlin M, Perez C, Howell JK (2005). Genome scale identification of *Treponema pallidum* antigens.. Infect Immun.

[23] Setubal JC, Reis M, Matsunaga J, Haake DA (2006). Lipoprotein computational prediction in spirochaetal genomes.. Microbiology.

[24] Fenno J, Müller KH, McBride BC (1996). Sequence analysis, expression, and binding activity of recombinant major outer sheath protein (Msp) of *Treponema denticola*.. J Bacteriol.

[25] Cameron CE, Lukehart SA, Castro C, Molini B, Godornes C (2000). Opsonic potential, protective capacity, and sequence conservation of the *Treponema pallidum* subspecies *pallidum* Tp92.. J Infect Dis.

[26] Brinkman MB, McGill MA, Pettersson J, Rogers A, Matejkova P (2008). A novel *Treponema pallidum* antigen, TP0136, is an outer membrane protein that binds human fibronectin.. Infect Immun.

[27] Liu H, Rodes B, George R, Steiner B (2007). Molecular characterization and analysis of a gene encoding the acidic repeat protein (Arp) of *Treponema pallidum*.. J Med Microbiol.

[28] Šmajs D, McKevitt M, Wang L, Howell JK, Norris SJ (2002). BAC library of *T. pallidum* DNA in *E. coli*.. Genome Res.

[29] Titz B, Rajagopala SV, Goll J, Häuser R, McKevitt MT (2008). The Binary protein interactome of *Treponema pallidum* - the syphilis spirochete.. PLoS One.

[30] Centurion-Lara A, Castro C, Barrett L, Cameron C, Mostowfi M (1999). *Treponema pallidum* major sheath protein homologue Tpr K is a target of opsonic antibody and the protective immune response.. J Exp Med.

[31] Centurion-Lara A, Godornes C, Castro C, Van Voorhis WC, Lukehart SA (2000a). The *tprK* gene is heterogeneous among *Treponema pallidum* strains and has multiple alleles.. Infect Immun.

[32] Centurion-Lara A, Sun ES, Barrett LK, Castro C, Lukehart SA (2000b). Multiple alleles of *Treponema pallidum* repeat gene D in *Treponema pallidum* isolates.. J Bacteriol.

[33] Centurion-Lara A, LaFond RE, Hevner K, Godornes C, Molini BJ (2004). Gene conversion: a mechanism for generation of heterogeneity in the *tprK* gene of *Treponema pallidum* during infection.. Mol Microbiol.

[34] Giacani L, Lukehart S, Centurion-Lara A (2007). Length of guanosine homopolymeric repeats modulates promoter activity of subfamily II tpr genes of *Treponema pallidum* ssp. *pallidum*.. FEMS Immunol Med Microbiol.

[35] Cox DL, Luthra A, Dunham-Ems S, Desrosiers DC, Salazar JC (2010). Surface immunolabeling and consensus computational framework to identify candidate rare outer membrane proteins of *Treponema pallidum*.. Infect Immun.

[36] Morozov V, Mushegian AR, Koonin EV, Bork P (1997). A putative nucleic acid-binding domain in Bloom's and Werner's syndrome helicases.. Trends in Biochem Sci.

[37] Bernstein DA, Keck JL (2003). Domain mapping of *Escherichia coli* RecQ defines the roles of conserved N- and C-terminal regions in the RecQ family.. Nucleic Acids Res.

[38] Stohl EA, Brockman JP, Burkle KL, Morimatsu K, Kowalczykowski SC (2003). *Escherichia coli* RecX inhibits RecA recombinase and coprotease activities in vitro and in vivo.. J Biol Chem.

[39] Stohl EA, Seifert HS (2001). The *recX* gene potentiates homologous recombination in *Neisseria gonorrhoeae*.. Mol Microbiol.

[40] Noonan JP, Grimwood J, Schmutz J, Dickson M, Myers RM (2004). Gene conversion and the evolution of protocadherin gene cluster diversity.. Genome Res.

[41] Harper KN, Liu H, Ocampo PS, Steiner BM, Martin A (2008). The sequence of the acidic repeat protein (*arp*) gene differentiates venereal from nonvenereal *Treponema pallidum* subspecies, and the gene has evolved under strong positive selection in the subspecies that causes syphilis.. FEMS Immunol Med Microbiol.

[42] Pallen MJ, Wren BW (2007). Bacterial pathogenomics.. Nature.

[43] Baseman JB, Nichols JC, Rumpp JW, Hayes NS (1974). Purification of *Treponema pallidum* from infected rabbit tissue: resolution into two treponemal populations.. Infect Immun.

[44] Zerbino DR, Birney E (2008). Velvet: Algorithms for de novo short read assembly using de Bruijn graphs.. Genome Res.

[45] Weinstock GM, Norris SJ, Sodergren E, Smajs D, Brogden KA, Roth JA, Stanton TB, Bolin CA, Minion FC, Wannemuehler MJ (2000). Identification of virulence genes in silico: infectious disease genomics.. Virulence mechanisms of bacterial pathogens.

[46] Nelson KE, Weinstock GM, Highlander SK, Worley KC, Creasy HH (2010). A Catalog of Reference Genomes from the Human Microbiome.. Science.

[47] Delcher A, Harmon D, Kasif S, White O, Salzberg SL (1999). Improved microbial gene identification with GLIMMER.. Nucleic Acids Res.

[48] Lukashin AV, Borodovsky M (1998). GeneMark.hmm: new solutions for gene finding.. Nucleic Acids Res.

[49] Gordon D, Abajian C, Green P (1998). Consed: A graphical tool for sequence finishing.. Genome Res.

[50] Librado P, Rozas J (2009). DnaSP v5: A software for comprehensive analysis of DNA polymorphism data.. Bioinformatics.

[51] Nei M, Kumar S (2000). Molecular Evolution and Phylogenetics..

[52] Tamura K, Dudley J, Nei M, Kumar S (2007). MEGA4: Molecular Evolutionary Genetics Analysis (MEGA) software version 4.0.. Mol Biol Evol.

