# Supplementary material for: Complete Genome Sequence of Treponema paraluiscuniculi, Strain Cuniculi A: The Loss of Infectivity to Humans Is Associated with Genome Decay
Source: PLoS One. 2011 May 31;6(5):e20415. doi: 10.1371/journal.pone.0020415 (PMC3105029; doi:10.1371/journal.pone.0020415)
Supplement: Table S1 — Genes fused in the T. paraluiscuniculi Cuniculi A genome when compared to the previously annotated T. pallidum subsp. pallidum Nichols genome [18] . (DOC) [file pone.0020415.s001.doc]

Table S1. Genes fused in the *T. paraluiscuniculi* Cuniculi A genome when compared to the previously annotated *T. pallidum* subsp. *pallidum* Nichols genome [18].

| **Nichols genes** | **Cuniculi A gene** | **Number of fused genes** |
| --- | --- | --- |
| TP0006, TP0007, TP0008 | TPCCA_0006 | 3 |
| TP0013, TP0014 | TPCCA_0013 | 2 |
| TP0018, TP0019 | TPCCA_0018 | 2 |
| TP0021, TP0022 | TPCCA_0021 | 2 |
| TP0172, TP0173 | TPCCA_0172 | 2 |
| TP0174, TP0175, TP0176 | TPCCA_0174 | 3 |
| TP0284, TP0285 | TPCCA_0284 | 2 |
| TP0286, TP0287 | TPCCA_0286 | 2 |
| TP0288, TP0289 | TPCCA_0288 | 2 |
| TP0299, TP0300 | TPCCA_0300 | 2 |
| TP0324, TP0325 | TPCCA_0324 | 2 |
| TP0377, TP0378 | TPCCA_0377 | 2 |
| TP0419, TP0420 | TPCCA_0419 | 2 |
| TP0433, TP0434 | TPCCA_0433 | 2 |
| TP0462, TP0463 | TPCCA_0462 | 2 |
| TP0468, TP0469 | TPCCA_0468 | 2 |
| TP0481, TP0482 | TPCCA_0481 | 2 |
| TP0587, TP0588 | TPCCA_0587 | 2 |
| TP0597, TP0598 | TPCCA_0597 | 2 |
| TP0702, TP0703 | TPCCA_0702 | 2 |
| TP0781, TP0782 | TPCCA_0781 | 2 |
| TP0859, TP0860 | TPCCA_0859 | 2 |
| TP0899, TP0900 | TPCCA_0899 | 2 |
| TP0928, TP0929 | TPCCA_0928 | 2 |
| TP1030, TP1031 | TPCCA_1031 | 2 |
