# Supplementary material for: Complete Genome Sequence of Treponema paraluiscuniculi, Strain Cuniculi A: The Loss of Infectivity to Humans Is Associated with Genome Decay
Source: PLoS One. 2011 May 31;6(5):e20415. doi: 10.1371/journal.pone.0020415 (PMC3105029; doi:10.1371/journal.pone.0020415)
Supplement: Table S2 — 99 genes encoding identical proteins in T. paraluiscuniculi Cuniculi A and T. pallidum subsp. pallidum Nichols genomes. (DOC) [file pone.0020415.s002.doc]

Table S2. 99 genes* encoding identical proteins in *T. paraluiscuniculi* Cuniculi A and *T. pallidum* subsp. *pallidum* Nichols genomes.

| **Gene** | **Gene function** | | **Gene name** | **Functional group** |
| --- | --- | --- | --- | --- |
| TPCCA_0060 | ribosomal protein L9 | | *rplI* | Translation |
| TPCCA_0063 | ribosomal protein S6 | | *rpsF* |  |
| TPCCA_0097 | initiation factor IF1 | | *infA* |  |
| TPCCA_0188 | ribosomal protein S10 | | *rpsJ* |  |
| TPCCA_0191 | ribosomal protein L23 | | *rplW* |  |
| TPCCA_0193 | ribosomal protein S19 | | *rpsS* |  |
| TPCCA_0194 | ribosomal protein L22 | | *rplV* |  |
| TPCCA_0196 | ribosomal protein L16 | | *rplP* |  |
| TPCCA_0198 | ribosomal protein S17 | | *rpsQ* |  |
| TPCCA_0200 | ribosomal protein L24 | | *rplX* |  |
| TPCCA_0206a | ribosomal protein L30 | | *rpmD* |  |
| TPCCA_0210 | ribosomal protein S13 | | *rpsM* |  |
| TPCCA_0237 | ribosomal protein L11 | | *rplK* |  |
| TPCCA_0243 | ribosomal protein S12 | | *rpsL* |  |
| TPCCA_0255 | ribosomal protein L31 | | *rpmE* |  |
| TPCCA_0362 | ribosomal protein L28 | | *rpmB* |  |
| TPCCA_0604 | ribosome recycling factor | | *frr* |  |
| TPCCA_0609 | asparagine--tRNA ligase | | *asnS* |  |
| TPCCA_0632 | tryptophan--tRNA ligase | | *trpS* |  |
| TPCCA_0682 | 23S rRNA methyltransferase | | *rrmJ* |  |
| TPCCA_0757 | peptide deformylase | | *def* |  |
| TPCCA_0758 | ribosomal protein S21 | | *rpsU* |  |
| TPCCA_0767 | elongation factor EF2 | | *fusA2* |  |
| TPCCA_0807 | ribosomal protein L32 | | *rpmF* |  |
| TPCCA_0830 | tRNA-pseudouridine synthase I | | *truA* |  |
| TPCCA_0848 | ribosomal protein L20 | | *rplT* |  |
| TPCCA_0887 | ribosomal protein S15 | | *rpsO* |  |
| TPCCA_0890 | ribosome-binding factor A | | *rbfA* |  |
| TPCCA_0909 | ribosomal protein L19 | | *rplS* |  |
| TPCCA_0951 | ribosomal protein L34 | | *rpmH* |  |
| TPCCA_1024 | ribosomal protein S9 | | *rpsI* |  |
| TPCCA_1025 | ribosomal protein L13 | | *rplM* |  |
| TPCCA_0078 | DegT/DnrJ/EryC1/StrS family pyridoxal dependent aminotransferase | |  | General metabolism |
| TPCCA_0080 | xanthine dehydrogenase | | *coxS* |  |
| TPCCA_0114 | probable HflC protein | | *hflC* |  |
| TPCCA_0168 | phosphoglycerate mutase family protein | |  |  |
| TPCCA_0291 | probable FMN-dependent family dehydrogenase | |  |  |
| TPCCA_0294 | ribose-phosphate diphosphokinase | | *prs* |  |
| TPCCA_0354 | dTMP kinase | | *tmk* |  |
| TPCCA_0476 | acetate kinase | | *ackA* |  |
| TPCCA_0509 | peroxiredoxin | | *ahpC* |  |
| TPCCA_0518 | thiamine pyrophosphokinase | |  |  |
| TPCCA_0595 | adenylate kinase | | *adk* |  |
| TPCCA_0602 | phosphatidate cytidylyltransferase | | *cdsA* |  |
| TPCCA_0615 | SUF system FeS assembly protein | |  |  |
| TPCCA_0794 | methionine adenosyltransferase | | *metK* |  |
| TPCCA_0817 | phosphopyruvate hydratase | | *eno* |  |
| TPCCA_0823 | probable superoxide reductase | | *sorA* |  |
| TPCCA_0885 | dUTP diphosphatase | | *dut* |  |
| TPCCA_0906 | probable RNA-binding protein | |  |  |
| TPCCA_0925 | nitrogenase (flavodoxin) | |  |  |
| TPCCA_0945 | ribulose-phosphate 3-epimerase | | *rpe* |  |
| TPCCA_0975 | probable tetrapyrrole methylase | |  |  |
| TPCCA_0982 | rhomboid family protein | |  |  |
| TPCCA_0991 | | rubredoxin |  |  |
| TPCCA_1027 | uridine phosphorylase | | *udp* |  |
| TPCCA_0106 | BCCT family betaine/carnitine/choline transporter | | *betT* | Transport |
| TPCCA_0119 | methionine ABC superfamily ATP binding cassette transporter, membrane protein | | *metI* |  |
| TPCCA_0120 | methionine ABC superfamily ATP binding cassette transporter, ABC protein | | *metN* |  |
| TPCCA_0235 | Sec family Type I general secretory pathway protein, subunit SecE | | *secE* |  |
| TPCCA_0292 | OOP family OmpA-OmpF porin | |  |  |
| TPCCA_0410 | RND superfamily resistance-nodulation-cell division protein:proton (H+) antiporter | | *secD* |  |
| TPCCA_0428 | two-sector ATPase, V(1) subunit D | | *ntpD1* |  |
| TPCCA_0528 | two-sector ATPase, V(1) subunit B | | *ntpB2* |  |
| TPCCA_0578 | Sec family Type I general secretory pathway protein | | *ftsY* |  |
| TPCCA_0589 | HPr family phosphotransferase system protein | | *hpr* |  |
| TPCCA_0725 | Mot family proton (H+) or sodium (Na+) transporter MotA | | *motA* |  |
| TPCCA_0964 | probable macrolide ABC superfamily ATP binding cassette transporter, ABC protein | |  |  |
| TPCCA_0396 | flagellar basal-body rod protein FlgB | | *flgB* | Flagellar biosynthesis |
| TPCCA_0403 | flagellar protein FliJ | | *fliJ* |  |
| TPCCA_0658 | flagellar assembly protein FliW | | *fliW* |  |
| TPCCA_0712 | probable flagellar synthesis regulator FleN | | *fleN1* |  |
| TPCCA_0722 | flagellar basal body-associated protein FliL | | *fliL2* |  |
| TPCCA_0726 | flagellar protein FlbD | | *flbD* |  |
| TPCCA_0792 | flagellar filament core protein FlaB | | *flaB1* |  |
| TPCCA_0853 | probable flagellar synthesis regulator FleN | | *fleN* |  |
| TPCCA_0868 | flagellar filament core protein FlaB | | *flaB2* |  |
| TPCCA_0943 | flagellar protein FliS | | *fliS* |  |
| TPCCA_0960 | flagellar basal body rod protein FlgG | | *flgG1* |  |
| TPCCA_0096 | probable DnaK suppressor protein | | *dksA* | Regulation |
| TPCCA_0167 | iron (Fe2+)/zinc (Zn2+)/manganese (Mn2+)-dependent transcriptional regulator | | *troR* |  |
| TPCCA_0236 | transcription antitermination protein NusG | | *nusG* |  |
| TPCCA_0365 | probable chemotaxis protein CheX | | *cheX* |  |
| TPCCA_0657 | carbon storage regulator | | *csrA* |  |
| TPCCA_1015 | transcription antitermination protein NusB | | *nusB* |  |
| TPCCA_0062 | single-strand DNA-binding protein | | *ssb1* | Cell processes, cell structure, DNA Replication, Repair, Recombination, Tanscription, Virulence |
| TPCCA_0071 | S14 family endopeptidase ClpB | | *clpB* |  |
| TPCCA_0072 | glutaredoxin-related protein | |  |  |
| TPCCA_0125 | exodeoxyribonuclease III | | *xthA* |  |
| TPCCA_0162 | crossover junction endoribonuclease subunit B | | *ruvB* |  |
| TPCCA_0216 | chaperone DnaK | | *dnaK* |  |
| TPCCA_0242 | DNA-directed RNA polymerase subunit beta prime | | *rpoC* |  |
| TPCCA_0390 | cell division protein FtsZ | | *ftsZ* |  |
| TPCCA_0541 | GTP-binding protein Era | | *era* |  |
| TPCCA_0626 | exonuclease SbcD | | *sbcD* |  |
| TPCCA_0649 | probable hemolysin | | *tlyC* |  |
| TPCCA_0701 | probable DNA-directed RNA polymerase subunit omega | | *rpoZ* |  |
| TPCCA_0768 | treponemal membrane protein A | | *tmpA* |  |
| TPCCA_0971 | tp34 lipoprotein | | *tpd* |  |

*35 genes encoding identical hypothetical or conserved hypothetical proteins in Cuniculi A and Nichols genomes are not shown (see Table S3).
