# Supplementary material for: Complete Genome Sequence of Treponema paraluiscuniculi, Strain Cuniculi A: The Loss of Infectivity to Humans Is Associated with Genome Decay
Source: PLoS One. 2011 May 31;6(5):e20415. doi: 10.1371/journal.pone.0020415 (PMC3105029; doi:10.1371/journal.pone.0020415)
Supplement: Table S3 — 35 genes of unknown function encoding identical proteins in T. paraluiscuniculi Cuniculi A and T. pallidum subsp. pallidum Nichols genomes. (DOC) [file pone.0020415.s003.doc]

Table S3. 35 genes of unknown function encoding identical proteins in *T. paraluiscuniculi* Cuniculi A and *T. pallidum* subsp. *pallidum* Nichols genomes.

| **Gene** | **Gene function** | **Gene expression [21]** |
| --- | --- | --- |
| TPCCA_0017 | conserved hypothetical protein | 0.882 |
| TPCCA_0041 | hypothetical protein | 1.413 |
| TPCCA_0047 | conserved hypothetical protein | 0.664 |
| TPCCA_0048 | conserved hypothetical protein | 1.14 |
| TPCCA_0070 | treponemal conserved hypothetical membrane protein | 0.617 |
| TPCCA_0148 | hypothetical membrane protein | 0.247 |
| TPCCA_0159 | treponemal conserved hypothetical protein | 1.004 |
| TPCCA_0214 | treponemal conserved hypothetical protein | 1.601 |
| TPCCA_0352 | conserved hypothetical protein | 1.183 |
| TPCCA_0421 | conserved hypothetical protein | 0.686 |
| TPCCA_0474 | conserved hypothetical protein | 3.366 |
| TPCCA_0480 | treponemal conserved hypothetical membrane protein | 0.624 |
| TPCCA_0490 | treponemal conserved hypothetical protein | 0.706 |
| TPCCA_0494 | conserved hypothetical protein | 2.035 |
| TPCCA_0552 | treponemal conserved hypothetical protein | 0.766 |
| TPCCA_0553 | conserved hypothetical membrane protein | 0.664 |
| TPCCA_0650 | conserved hypothetical protein – translation [29] | 0.376 |
| TPCCA_0676 | hypothetical protein | 0.949 |
| TPCCA_0679 | treponemal conserved hypothetical membrane protein | 0.73 |
| TPCCA_0690 | treponemal conserved hypothetical protein | 0.371 |
| TPCCA_0700 | treponemal conserved hypothetical protein | 3.725 |
| TPCCA_0744 | conserved hypothetical protein | 1.638 |
| TPCCA_0772 | treponemal conserved hypothetical protein – gene regulation [29] | 1.12 |
| TPCCA_0784 | treponemal conserved hypothetical protein | 0.574 |
| TPCCA_0816 | treponemal conserved hypothetical protein | 0.69 |
| TPCCA_0869 | hypothetical protein | 13.54 |
| TPCCA_0874 | conserved hypothetical protein | 0.703 |
| TPCCA_0875 | conserved hypothetical protein | 1.14 |
| TPCCA_0911 | conserved hypothetical protein | 0.701 |
| TPCCA_0914 | conserved hypothetical protein | 0.607 |
| TPCCA_0941 | treponemal conserved hypothetical protein – flagellar biosynthesis [29] | 1.558 |
| TPCCA_0942 | treponemal conserved hypothetical protein | 1.122 |
| TPCCA_0974 | treponemal conserved hypothetical protein | 3.856 |
| TPCCA_0992 | conserved hypothetical protein | 3.294 |
| TPCCA_1032 | conserved hypothetical protein | nd |

nd, not determined
