# Supplementary material for: Complete Genome Sequence of Treponema paraluiscuniculi, Strain Cuniculi A: The Loss of Infectivity to Humans Is Associated with Genome Decay
Source: PLoS One. 2011 May 31;6(5):e20415. doi: 10.1371/journal.pone.0020415 (PMC3105029; doi:10.1371/journal.pone.0020415)
Supplement: Table S4 — T. paraluiscuniculi Cuniculi A genes with unknown cell function containing internal frameshifts and/or major sequence changes (MSC) compared to the Nichols orthologs. (DOC) [file pone.0020415.s004.doc]

Table S4. *T. paraluiscuniculi* Cuniculi A genes with unknown cell function containing internal frameshifts and/or major sequence changes* (MSC) compared to the Nichols orthologs.

| **Gene** | **Protein prediction*^a^*** | **Type of change** | **Gene expression*^b^*** | **Described molecular interaction of Nichols orthologs with other proteins with known function [28]** | **Ka/Ks ratio (if applicable) and**  **estimation of selection type*^c^*** | **Remark/reference** |
| --- | --- | --- | --- | --- | --- | --- |
| TPCCA_0012 | HP | frameshift mutation | *0.681* | TP0500 (*pbp-1*) |  |  |
| TPCCA_0031 | HP | MSC | *2.156* |  | NA (divided by 0) positive selection |  |
| TPCCA_0126 | CHP | frameshift mutation | *1.115* |  |  |  |
| TPCCA_0127-129*^c^* | HP | genes deleted | *1.523, 0.702, 0.763* | TP0127: TP0330 (cell division protein, putative) |  | [2] |
| TPCCA_0132 | HP | frameshift mutation | *0.832* |  |  | [2] |
| TPCCA_0133*^d^* | HP | MSC | *2.093* | TP0640 (*mcp2-3*) | 1.00 neutral selection | [2] |
| TPCCA_0134-135 | HP | genes deleted | *3.586,1.526* | TP0588 (putative DNA polymerase III), TP0965 (membrane fusion protein, putative) |  | [2] |
| TPCCA_0137 | HP | MSC | *1.94* |  | 1.69 neutral selection | [2] |
| TPCCA_0180 | HP | missing start codon, frameshift mutation | *1.741* |  |  |  |
| TPCCA_0245 | CHP | MSC | *0.694* |  | 0.81 neutral selection |  |
| TPCCA_0304 | TCHP | MSC | *1.235* |  | 0.46 purifying selection |  |
| TPCCA_0311 | HP | frameshift mutation | *1.398* |  |  | [2] |
| TPCCA_0315*^d^* | CHP | MSC | *0.919* |  | 0.29 purifying selection | [2] |
| TPCCA_0318 | HP | frameshift mutation | *0.947* |  |  | [2] |
| TPCCA_0347 | HMP | internal stop codon | *1.658* |  | 0.40 neutral selection |  |
| TPCCA_0355 | HP | frameshift mutation | *0.556* |  |  |  |
| TPCCA_0370 | HP | MSC | *0.822* |  | NA (divided by 0) neutral selection |  |
| TPCCA_0462*^d,e^* | CHP | MSC | *4.894, 4.184* | TP0684 (methylgalactoside transport system substrate-binding protein), TP1013 (*groES*), TP0948 (*jag*), TP0271 (*parB*) | 1.21 neutral selection | TP0462 and TP0463 genes are fused also in the Nichols genome, [26] |
| TPCCA_0470*^c^* | CHP | partial deletion | *3.103* | TP0343 (A/G-specific adenine glycosylase, putative) | 0.72 neutral selection | [2] |
| TPCCA_0479 | HMP | frameshift mutation | *2.736* |  |  |  |
| TPCCA_0487 | TCHP | MSC | *1.181* | TP0380 (DNA repair helicase, putative) | 1.35 neutral selection | [2] |
| TPCCA_0515 | CHOMP | MSC | *0.104* | TP0628 (putative nicotinate phosphoribosyltransferase) | 1.45 neutral selection |  |
| TPCCA_0548 | TCHMP | MSC | *0.898* |  | 1.33 neutral selection |  |
| TPCCA_0577 | TCHMP | MSC | *0.531* |  | 1.05 neutral selection |  |
| TPCCA_0594 | CHP | frameshift mutation | *2.238* |  |  |  |
| TPCCA_0618*^c^* | TCHP | MSC | *1.227* | TP0779 (*dedA*), TP0688 (*mccF*), TP0006 (tp75 protein), TP0946 (*cyp*), TP634 (*lig*) | 1.49 neutral selection | [2] |
| TPCCA_0619 | TCHP | gene deleted | *1.068* | TP0755 (PTS system, nitrogen regulatory IIA component, *ptsN-2*) |  | [2] |
| TPCCA_0651 | CHMP | partial deletion at 5`end – start codon missing | *0.682* | TP0397 (*flgC*), TP0398 (*fliE*), TP0586 (*leuS*), TP1005 (*dnaH*), TP1041 (*recR*) | 0.33 purifying selection |  |
| TPCCA_0698 | HMP | internal stop codon | *1.813* | TP0582 (conserved hypothetical integral membrane protein) | 0.41 neutral selection |  |
| TPCCA_0699 | HP | frameshift mutation | *2.477* | TP0233 (anti-sigma F factor antagonist) |  |  |
| TPCCA_0707 | HMP | frameshift mutation | *0.557* |  |  |  |
| TPCCA_0795 | HP | frameshift mutation | *0.921* | TP1038 (bacterioferrin, TpF1), TP727 (*flgE*), TP0080 (quinoline 2-oxidoreductase), TP0946 (*gidB*) |  |  |
| TPCCA_0818 | HP | frameshift mutation | *1.21* | TP0112 (*pepC*) |  |  |
| TPCCA_0856 | TCHP | MSC | *5.697* | TP0398 (*fliE*),TP0870 (*flaB*) | 0.66 neutral selection |  |
| TPCCA_0857 | HP | frameshift mutation | *4.339* |  |  |  |
| TPCCA_0858 | TCHP | MSC | *11.77* | TP1005 (*dnaH*) | 0.70 neutral selection |  |
| TPCCA_0859*^e^* | TCHP | MSC | *2.231,1.204* |  | 0.99 neutral selection | [2] |
| TPCCA_0865*^d^* | TCHP | MSC | *0.371* |  | 1.37 neutral selection | [2] |
| TPCCA_0895*^d^* | TCHP | insertions at 5`end – start codon missing, frameshift mutation | *0.952* | TP0236 (*nusG*), TP0233 (anti-sigma F factor antagonist, putative) |  |  |
| TPCCA_0896*^d^* | HP | frameshift mutation | *1.864* | TP0236 (*nusG*), TP0233 (anti-sigma F factor antagonist, putative) |  |  |
| TPCCA_0918 | CHMP | frameshift mutation | *5.759* |  |  |  |
| TPCCA_0922 | HP | frameshift mutation | *1.599* |  |  |  |
| TPCCA_0923 | HOMP | MSC | *0.54* |  | 0.43 purifying selection |  |
| TPCCA_0950 | HP | frameshift mutation | *0.998* |  |  |  |
| TPCCA_0967 | TCHP | MSC | *2.844* |  | 0.60 neutral selection | [2] |
| TPCCA_0968 | TCHP | MSC | *3.256* |  | 1.10 neutral selection | [2] |
| TPCCA_0969 | TCHOMP | MSC | *2.751* |  | 0.44 neutral selection |  |
| TPCCA_0970 | HP | frameshift mutation | *2.77* |  |  |  |
| TPCCA_0976 | HMP | frameshift mutation | *2.751* | TP0701 (DNA-directed RNA polymerase, putative) |  |  |

*Major sequence changes were defined as continuous amino acid replacements comprising 10 and more residues or 20 and more dispersed amino acid replacements. Annotations of the Cuniculi A genes predicting longer proteins at the N-terminus with existing potential downstream start codons at corresponding positions as in the Nichols genome were not considered as major sequence changes.

*^a^*HP, hypothetical protein; CHP, conserved hypothetical protein; HMP, hypothetical membrane protein; TCHP, treponemal conserved hypothetical protein; CHOMP,conserved hypothetical outer membrane protein; TCHMP, treponemal conserved hypothetical membrane protein; CHMP, conserved hypothetical membrane protein; HOMP, hypothetical outer membrane protein; TCHOMP, treponemal conserved hypothetical outer membrane protein.

*^b^*gene expression levels from Šmajs et al. [21]

^c^Ka/Ks ratios were calculated by the MEGA4 software [52] and the selection test was calculated using the Kumar model [51]

*^d^*corresponding protein identified as antigen [22]

*^e^*fused genes (see Table S1)
